# Real time cardiac MRI and its clinical usefulness in arrhythmias and wall motion abnormalities

**DOI:** 10.1186/1532-429X-16-S1-P34

**Published:** 2014-01-16

**Authors:** Christina Unterberg-Buchwald, Martin Fasshauer, Jan M Sohns, Wieland Staab, Andreas Schuster, Dirk Voit, Johannes T Kowallick, Michael Steinmetz, Jens Frahm, Joachim Lotz

**Affiliations:** 1 Cardiology, University Clinic Goettingen, Goettingen, Germany; 2 Institute of Diagnostic and Interventional Radiology, University Clinic Goettingen, Goettingen, Germany; 3 Biomedizinische NMR ForschungsGmbH, Max-Planck-Institut für biophysikalische Chemie, Goettingen, Germany; 4 Pediatric Cardiology and Intensive Care, University Clinic Goettingen, Goettingen, Germany

## Background

Analysis of cardiac function in patients with arrhythmias is very limited or nearly impossible in ECG synchronized cine acquisitions with balanced SSFP. A real-time method at 1.5 T with SSFP contrast was used to show that this method is superior for image quality and analysis of ventricular function in a subset of patients with atrial fibrillation and AV-Block.

## Methods

Radial gradient-echo sequences with fully balanced SSFP gradients and at least 15-fold undersampling (real-time SSFP, RT) and conventional ECG-synchronized cine SSFP CMR (Cine) was used on a 1.5 T scanner system. Patients who had permanent arrhythmias (most often atrial fibrillation, AA; n = 8) or wall motion abnormalities (WMA; n = 3) were scanned in the standard views and compared to patients in sinus rhythm (SR;n = 21) without wall motion abnormalities. Image reconstruction of RT was performed offline by regularized nonlinear inversion. Quality (IQ) of scan was detected using an image quality score (ranging from 0 = no diagnostic quality to 1 = reduced diagnostic quality, 2 = many artifacts, 3 = some artifacts, and 4 = optimal diagnostic quality). Cardiac function was analyzed using a semiautomatic contour detection (Q mass, Medis, NL) applied to 5 consecutive baets in RT and Cine avering data of 10-12 beats. All analysis was done by two blinded observers (3 and 8 years experience of CMR evaluation).

## Results

IQ was comparable for Cine and RT SSFP in patients with WMA or patients in sinus rhythm without WMA given in Table 1. However, IQ was significantly better for RT compared to Cine in all three short axis in patients with arryhthmias. Functional parameters showed no significant differences, however there was a trend to lower values for enddiastolic, endsystolic and beat volumes for Cine (10-12 beats) compated to RT (5 consecutive beats) in RT.

**Table 1:** 

Views for IQ and quantitative parameters	Patient groups	Number of views	RT	Cine
Short axis base (IQ)	AA WMA SR	16 6 42	3.94 ± 0.25 * 4.00 ± 0.00 3.62 ± 0.54	2.75 ± 0.68 4.00 ± 0.00 3.95 ± 0.22
Short axis mid (IQ)	AA WMA SR	16 6 42	3.81 ± 0.40 * 3.67 ± 0.5 3.48 ± 0.67	2.69 ± 0.60 4.00 ± 0.00 3.93 ± 0.26

Short axis apex (IQ)	AA WMA SR	16 6 42	3.81 ± 0.40 * 3.00 ± 1.26 3.07 ± 0.97	2.19 ± 0.91 3.50 ± 0.55 3.70 ± 0.55

Enddiastolic volume [ml]	AA WMA SR	16 3 16	55.15 ± 10.17 52.39 ± 3.91 47.50 ± 12.70	36.80 ± 20.13 43.10 ± 6.73 51.32 ± 18.4
Endsystolic volume [ml]	AA WMA SR	16 3 16	32.30 ± 13.11 30.50 ± 9.80 25.10 ± 13.0	21.60 ± 16.50 24.10 ± 6.86 22.40 ± 10.70

Beat volume [ml]	AA WMA SR	16 3 16	22.83 ± 6.30 21.90 ± 8.50 22.37 ± 5.30	15.20 ± 6.20 19.00 ± 1.27 29.84 ± 16.7

Ejection fraction [%]	AA WMA SR	16 3 16	43.50 ± 15.10 41.70 ± 16.70 50.00 ± 12.20	46.30 ± 16.10 45.00 ± 6.90 57.90 ± 12.50

* p < 0.001 RT vs Cine

## Conclusions

RT cardiac MRI is a robust method with high image quality that has the potential to allow functional analysis of sequences in patients with arrhythmias that are often difficult or impossible to analyze by cine SSFP.

## Funding

No funding.

